# Contraception, fertility and inflammatory bowel disease (IBD): a survey of the perspectives of patients, gastroenterologists and women’s healthcare providers

**DOI:** 10.1136/bmjgast-2024-001669

**Published:** 2025-03-16

**Authors:** Guillaume Le Cosquer, Cyrielle Gilletta, Florian Béoletto, Barbara Bournet, Louis Buscail, Emmeline di Donato

**Affiliations:** 1 Department of Gastroenterology and Pancreatology, University Hospital Centre Toulouse, Toulouse, Occitanie, France; 2 Department of Gynecology and Obstetrics, University Hospital Centre Toulouse, Toulouse, Occitanie, France

**Keywords:** INFLAMMATORY BOWEL DISEASE, QUALITY OF LIFE, Life Style, IBD CLINICAL

## Abstract

**Objective:**

Despite guidelines indicating no contraindications for contraceptives in women with inflammatory bowel disease (IBD), this population shows increased voluntary childlessness and lower contraceptive use. Knowledge gaps among healthcare providers on IBD’s impact on fertility and contraception may drive these trends. This survey assessed knowledge discrepancies among IBD patients, gastroenterologists (GEs), and women’s healthcare providers (WHPs) regarding fertility and contraception.

**Methods:**

An anonymous survey was conducted between August and December 2023, targeting IBD patients of childbearing age, GEs and WHPs. The questionnaire was offered consecutively to all patients consulting or hospitalised in our department. Additionally, the survey link was shared with healthcare professionals during dedicated training sessions. It assessed awareness of IBD-related fertility and contraception impacts.

**Results:**

Two hundred twenty-two participants fulfilled the survey (100 patients, 50 GEs and 72 WHPs). Among patients (63% with Crohn’s disease), 95% were on biologic or immunosuppressant therapy. Nearly half (47%) of women had not discussed fertility or contraception with their GE, and only 22% had done so on request. A majority (80% of women, 54% of GEs) were unsure if IBD affects contraception efficacy, and 50% of WHPs believed oral contraceptives to be less effective for IBD patients. Key concerns influencing patients’ fertility decisions included the impact of IBD medication on pregnancy (51%), risk of passing IBD to offspring (47%) and potential flare-ups during pregnancy (39%).

**Conclusion:**

Significant knowledge gaps on fertility and contraception in IBD persist among patients, GEs and WHPs.

**WHAT IS ALREADY KNOWN ON THIS TOPIC**

- Patients with inflammatory bowel disease (IBD) are more prone to voluntary childlessness compared with healthy individuals, partly due to insufficient knowledge about IBD’s impact on fertility.
- Limited data exist on the level of awareness among healthcare professionals and patients regarding the influence of IBD on contraception.

**WHAT THIS STUDY ADDS**

- Our findings confirm that discussions on IBD’s impact on contraceptive methods are infrequently initiated by gastroenterologists (GEs) and that false beliefs persist among both IBD patients and GEs.
- While IBD patients frequently engage in discussions about contraception and fertility with women’s healthcare providers, these providers typically lack IBD-specific knowledge.

**HOW THIS STUDY MIGHT AFFECT RESEARCH, PRACTICE OR POLICY**

- This study highlights the urgent need for enhanced interdisciplinary communication and targeted continuing medical education (CME) programmes focusing on IBD-specific contraception and fertility. Such initiatives are essential for empowering IBD patients to make informed reproductive health decisions.

## Introduction

 Inflammatory bowel diseases (IBD) are lifelong conditions primarily affecting young adults, often during their reproductive years, with peak diagnoses occurring between the ages of 20 and 40.^1^ Advances in IBD management, particularly with the early introduction of immunosuppressants and biologics, have significantly altered disease progression.^2^ Recent European guidelines recommend maintaining ongoing IBD therapy during pregnancy to ensure disease control and initiating new treatments as necessary for flare management during pregnancy.^3^ However, concerns regarding the effects of IBD and its treatments on fertility, pregnancy outcomes and offspring health often impact patient decisions around family planning.^4^

A meta-analysis by Tavernier *et al* found that misconceptions about IBD’s effects on reproduction contribute to high rates of voluntary childlessness (17%–38%) among patients, which in turn lowers fertility rates.^5^ Although research suggests that gastroenterologists (GEs) and IBD nurses generally possess good theoretical knowledge of IBD’s effects on fertility and pregnancy, patient education appears insufficient in real-world settings.^6 7^ Studies indicate that fewer than half of IBD patients receive preconception counselling, despite its association with lower rates of voluntary childlessness.^8 9^ In response, recent ECCO guidelines emphasise the importance of providing comprehensive education on pregnancy and family planning for all women with IBD of reproductive age.^3^

Additionally, reducing unplanned pregnancy is essential, as achieving disease remission before pregnancy and avoiding contraindicated medications are critical for maternal and foetal health. Active IBD during pregnancy increases the risk of miscarriage, stillbirth, preterm labour and intrauterine growth restriction.^10^ Yet, contraceptive use among women with IBD remains lower than in the general population,^11^ with prior surgeries and biologic therapy linked to lower contraceptive use.^12^ Studies show that GEs rarely discuss contraception with IBD patients, likely due to knowledge gaps among GEs and women’s healthcare providers (WHPs; defined as obstetricians, gynaecologists and midwives) regarding IBD’s effects on contraception.^13^

This study aims to assess the knowledge of women with IBD, WHPs and GEs regarding the impact of IBD on fertility and contraception.

## Methods

### Study design

An anonymous web cross-sectional survey was conducted from August to December 2023 using Sphinx online software. It targeted women of childbearing age with IBD who were receiving care at a tertiary IBD centre, including both outpatient and inpatient settings. The survey was distributed to consecutive patients on the day of their visit, with an arbitrary maximum limit of 100 participants, at which point the survey was closed. Concurrently, similar surveys were distributed via email to GEs and WHPs who were either registered for Continuing Medical Education programmes or involved in multidisciplinary IBD consultations (CROSS checklist available as online supplemental material 1). While some practitioners who completed the survey may have been employed at the same hospital where the surveyed women were being treated, the anonymity of the responses prevented the cross-referencing of their respective answers. To address non-response error, multiple reminders were sent to encourage completion of the questionnaire, and the non-response rate was tracked and recorded.

### Data collection

Patient data included demographics (age, height, weight, BMI, smoking status), IBD type (ulcerative colitis, Crohn’s disease or indeterminate colitis), disease classification (location and extension per Montreal criteria^14^), disease duration, prior IBD surgeries, extraintestinal manifestations and IBD treatment exposure (aminosalicylates, steroids, immunosuppressants, biologics or Janus Kinase (JAK) inhibitors). Assistance from healthcare professionals was provided to participants for completing questionnaire sections related to IBD characteristics. Patient-reported outcomes on IBD activity (self-evaluated on a scale of 0 to 10 to ensure ease and quick completion), fertility and contraception knowledge were assessed (online supplemental material 2).

WHPs and GEs provided demographic information, including age, gender, specialisation, practice setting, years of experience and IBD patient caseload. They were also surveyed on their knowledge of IBD-related fertility and contraception (as detailed in onlinesupplemental materials 3  4). Only fully completed surveys were included in the analysis.

### Ethical considerations

The study was conducted in accordance with the principles of good clinical practice and the 1975 Declaration of Helsinki at all times. According to the French ethic and regulatory law (public health code), prospective studies using anonymised data do not require submission to an ethical committee, but they have to be declared or covered by reference methodology of the French National Commission for Informatics and Liberties (CNIL). A collection and computer processing of personal and medical data was implemented to analyse the results of the research. After evaluation and validation by the data protection officer and according to the General Data Protection Regulation, this study, completing all the criteria, is recorded in the register of retrospective studies of the Toulouse University Hospital (RnIPH 2021–143) and covered by the MR-004 (CNIL number: 2 206 723 v 0). This study was approved by Toulouse University Hospital and confirms that ethical requirements were totally respected in the above report. All participants received information on data usage and the right to opt out; survey continuation indicated informed consent.

### Statistical analysis

Continuous variables are expressed as median and IQR (or mean and SD), and categorical variables are expressed as proportion and percentage. To evaluate the confounding effect of taking care (or not) of women with IBD on WHP answers, a comparison was drawn by a χ2 test (or Fisher’s exact tests when appropriate) between the two groups. Two-sided statistical tests have been used for all analyses, and a p value<0.05 has been considered significant. The statistical analyses have been performed with the IBM SPSS Statistics software (V.29.0.1.0).

## Results

### Population characteristics

Of the approximately 200 reproductive-age women with IBD to whom the survey was distributed, 100 participated (63 with Crohn’s disease, 35 with ulcerative colitis, and two with indeterminate colitis). The median age at inclusion was 34 years (IQR 26–42), and the median duration of IBD was 8.5 years (IQR 3–12). In terms of disease activity, 53% of the respondents considered themselves to be in clinical remission, with a median self-reported disease activity score of 3 on a 0–10 scale (IQR 0–5). Additionally, 95% of the participants had been exposed to at least one biologic or immunosuppressant during their disease course, while 16% had undergone surgical intervention for IBD (14 ileocaecal resections, one small bowel resection and one total proctocolectomy with ileal pouch-anal anastomosis). A comprehensive summary of these characteristics is presented in table 1.

**Table 1:** Patient characteristics

	Patients (n=100)
Crohn’s disease location, n (%)*	
Ileal (L1)	22 (34.9)
Colonic (L2)	11 (17.5)
Ileocolonic (L3)	25 (39.7)
Upper GI tract (L4)	5 (7.9)
Crohn’s disease behaviour, n (%)*	
Non-stricturing, non-penetrating (B1)	12 (19)
Stricturing (B2)	29 (46)
Penetrating (B3)	22 (35)
Perianal lesions, n (%)*	19 (31)
Surgical drainage of perianal abscesses, n (%)*	19 (31)
**Colitis location, n (%)†**	
Proctitis (E1)	4 (11)
Left-sided colitis (E2)	20 (54)
Pancolitis (E3)	13 (35)
**Extra-intestinal manifestations, n (%**)	37 (37)
Arthritis	12 (12)
Skin disease	20 (20)
Uveitis	3 (3)
Primary sclerosing cholangitis	4 (4)
**Current therapeutic line, n (%**)	
Aminosalicylates	12 (12)
Steroids	10 (10)
Thiopurine	18 (18)
Methotrexate	4 (4)
Ciclosporin	2 (2)
Infliximab	37 (37)
Adalimumab	17 (17)
Golimumab	7 (7)
Certolizumab pegol	1 (1)
Ustekinumab	8 (8)
Guselkumab	2 (2)
Vedolizumab	14 (14)
**Previous IBD treatments, n (%**)	
Aminosalicylates	50 (50)
Steroids	79 (79)
Thiopurine	56 (56)
Methotrexate	13 (13)
Ciclosporin	1 (1)
Infliximab	26 (26)
Adalimumab	31 (31)
Golimumab	3 (3)
Ustekinumab	8 (8)
Vedolizumab	13 (13)
Tofacitinib	7 (7)
**Mean BMI (SD**)	24.4 (7.3)
**Smoking status, n (%**)	
Non-smoker	56 (56)
Former smoker	24 (24)
Active smoker	20 (20)

* Among 63 women with Crohn’s disease

† Among 35 women with ulcerative colitis and two indeterminate colitis

IBD, inflammatory bowel diseases.

The questionnaire was distributed to approximately 150 GEs, with 50 completing it (23 men and 27 women, representing a response rate of 33.3%). Of these, seven (14%) worked in general hospitals, 15 (26%) in private medical practices, 25 (50%) in university hospitals (including 12 residents) and three (10%) had dual public and private practices. The median number of years of IBD experience among GEs was 8 years (IQR 4–16). Self-reported data indicated that IBD patients constituted a median of 10% of their overall patient base (IQR 5–20). Only five GEs (10%) had participated in specialised continuing medical education (CME) or training focused on contraception, fertility, and IBD.

The survey was also sent to approximately 650 WHPs, with 72 responding (three men and 69 women; response rate: 11%). The respondents included eight medical gynaecologists (11.1%, including four residents), 15 obstetricians (20.8%, including four residents) and 49 midwives (68.1%). One WHP (1.4%) worked in general hospitals, 20 (28%) in private medical practices, 47 (65%) in university hospitals and four (5.6%) had dual public and private practices. The median number of years of medical experience for WHPs was 10 years (IQR 3–17). Notably, 50 WHPs (69%) reported prior experience caring for women with IBD, but only two (2.8%) had attended CME or training specifically on contraception, fertility, and IBD.

### Patient information on contraception and fertility

**Figure 1:**
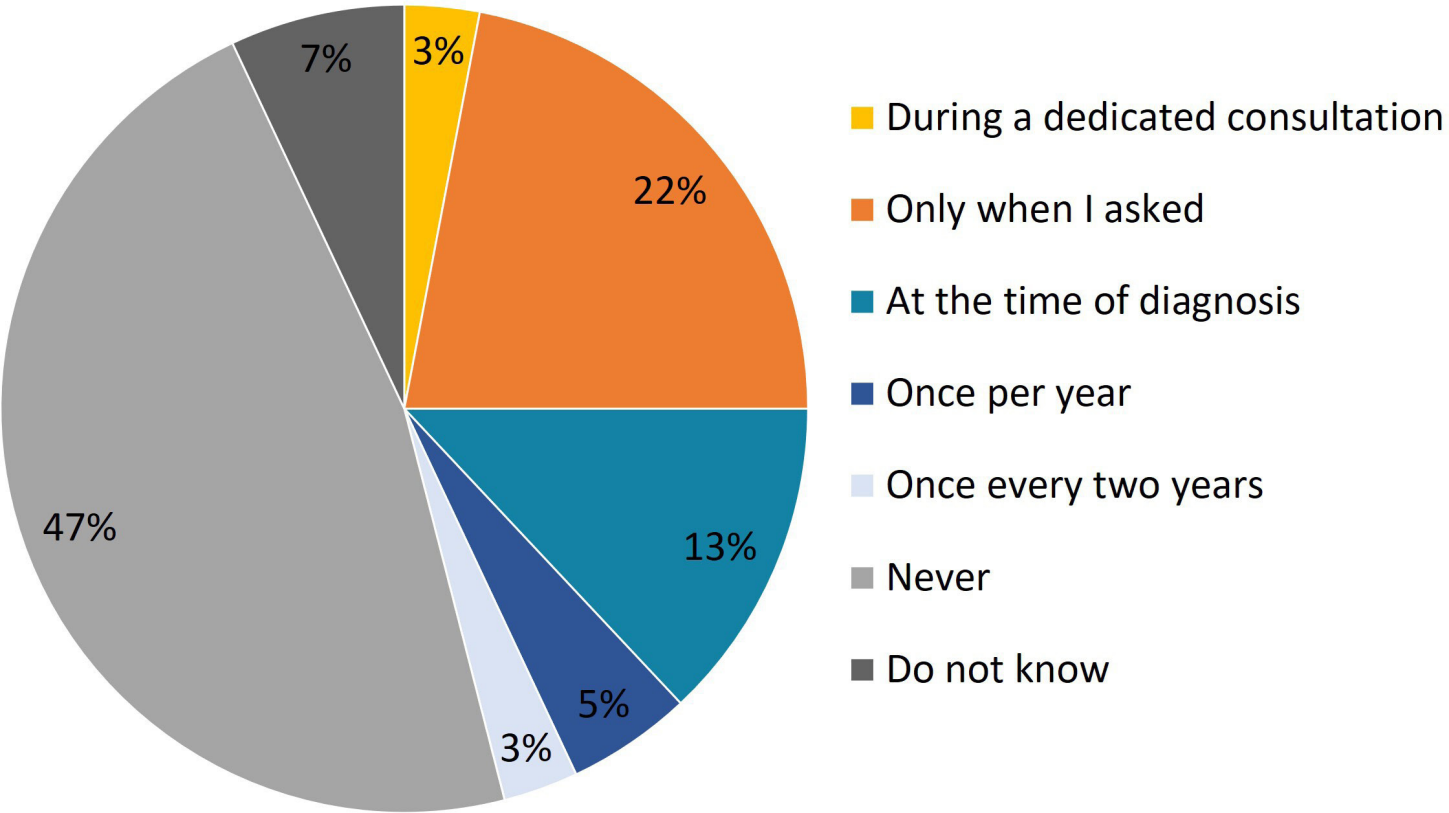
Frequency of inflammatory bowel diseases (IBD)-specific discussions on fertility and contraception. Patient responses to the question, ‘how often has your gastroenterologist discussed the impact of IBD on fertility and contraception with you?’ (in percentages).

A total of 34 women (34%) reported having concerns about the potential impact of their disease on their contraceptive choices. The majority of respondents indicated that discussions with their GEs about the impact of IBD on fertility and contraception either did not take place or were initiated by the patient (figure 1). This was corroborated by 27 GEs (54%), who acknowledged that they only addressed these topics on patient request. In contrast, 10 GEs (20%) discussed these issues at the time of diagnosis, five (10%) once a year, three (6%) every 2 years, one (2%) during a dedicated visit, while three (6%) admitted to never having these discussions, and (2%) was unsure if they had.

When women with IBD were asked which healthcare professionals they believed could answer questions about the impact of IBD on contraception and fertility, their responses were: medical gynaecologists (75%), obstetricians (71%), GEs (52%), midwives (42%) and general practitioners (22%). In response to the same question, GEs identified GEs (86%), medical gynaecologists (82%), obstetricians (64%), midwives (54%) and general practitioners (22%) as suitable sources of information. WHPs answered similarly, naming obstetricians (90.3%), GEs (87.5%), medical gynaecologists (76.4%), general practitioners (43.1%), and midwives (25%).

### Birth control contraception

**Figure 2:**
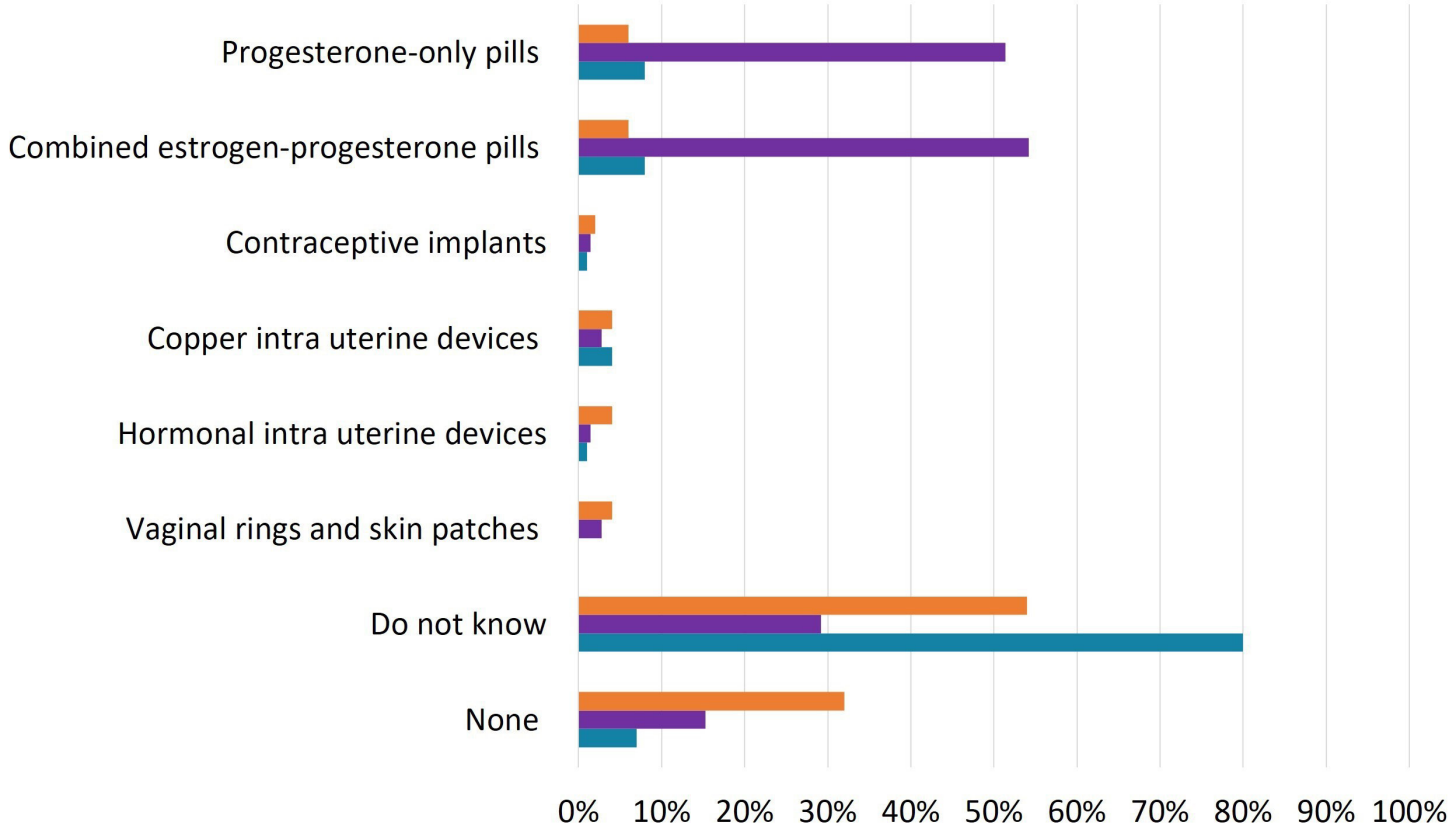
Contraceptive methods with suspected reduced effectiveness in women with inflammatory bowel disease (IBD) patient (green), gastroenterologist (orange) and women’s healthcare provider (purple) responses to the question, ‘which of the following contraceptive methods might be less effective in patients with IBD, not considering any effects from IBD treatments?’ (in percentages).

Concerning contraceptive methods, 80 women with IBD (80%) reported that they were unsure whether IBD could reduce the effectiveness of any contraceptive methods. Similarly, 27 GEs (54%) shared the same uncertainty. In contrast, half of WHPs believed that oral contraceptives, whether progesterone-only or combined oestrogen-progesterone pills, had reduced effectiveness in the context of IBD (detailed results in figure 2).

Additionally, 72 women (72%) stated that they did not know if any contraceptive method could trigger IBD flare-ups. Of the remainder, 12 women (12%) identified combined oestrogen-progesterone pills as potentially causing flare-ups, while eight (8%) pointed to progesterone-only pills, four (4%) to copper intrauterine devices (IUDs), three (3%) to hormonal IUDs and two (2%) to vaginal rings and skin patches. Only 11 women (11%) believed that none of the contraceptive methods posed a risk of IBD flare-ups.

GEs’ opinions were divided: 20 (40%) stated that none of the contraceptive methods posed a risk of IBD flare-ups, while 22 (44%) admitted they did not know if any were associated with increased risk. The remaining GEs believed that specific methods, such as combined oestrogen-progesterone pills (14%), contraceptive implants (2%), hormonal IUDs (8%), and vaginal rings and skin patches (4%), could be linked to an increased risk of flare-ups.

Among WHPs, 38 (52.8%) indicated they did not know if any contraceptive methods carried a risk of IBD flare-ups. Only eight (11.1%) believed that none posed a risk. The remaining WHPs identified combined oestrogen-progesterone pills (23.6%), copper IUDs (19.4%), vaginal rings and skin patches (9.7%), progesterone-only pills (5.6%) and hormonal IUDs (4.2%) as potential triggers. WHPs who had experience with IBD patients were significantly less likely to report that copper IUDs posed a risk of IBD flare-ups compared with those without such experience (10% vs 40.9%; p=0.002).

### Emergency contraception

**Figure 3:**
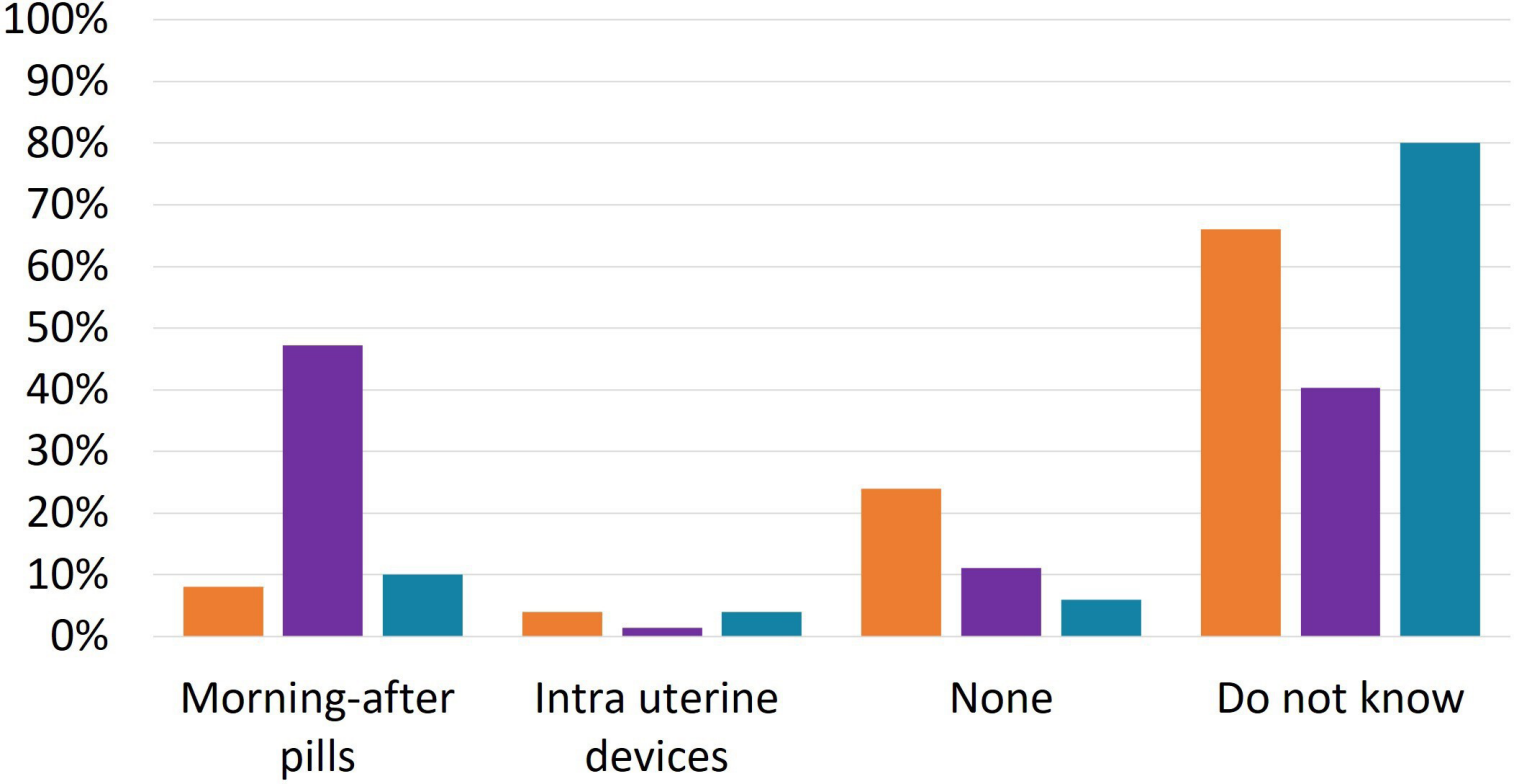
Emergency contraceptive methods with suspected reduced effectiveness in women with inflammatory bowel disease (IBD) patient (green), gastroenterologist (orange) and women’s healthcare provider (purple) responses to the question, ‘which emergency contraceptive methods, if any, may have reduced effectiveness in IBD patients, not considering any effects from IBD treatments?’ (in percentages).

Among WHPs, 47.2% believed that the effectiveness of morning-after pills was reduced in women with IBD, whereas the majority of patients and GEs were unsure if any emergency contraceptive methods were less effective in the context of IBD (figure 3). When asked if any emergency contraceptive methods were contraindicated for women with IBD, responses were similar across groups: 8% of patients, 6% of GEs and 8.3% of WHPs indicated morning-after pills; 7% of patients, 6% of GEs and 11.1% of WHPs identified IUDs. A significant proportion, however, responded that none of the methods were contraindicated (7% of patients, 36% of GEs and 34.7% of WHPs). The majority in each group reported uncertainty. Notably, WHPs with experience caring for women with IBD were significantly less likely to report that IUDs were contraindicated (2% vs 31.8%; p<0.001) and morning-after pills less effective (38% vs 68.2%; p=0.018).

### Fertility rate and medically assisted procreation

**Figure 4:**
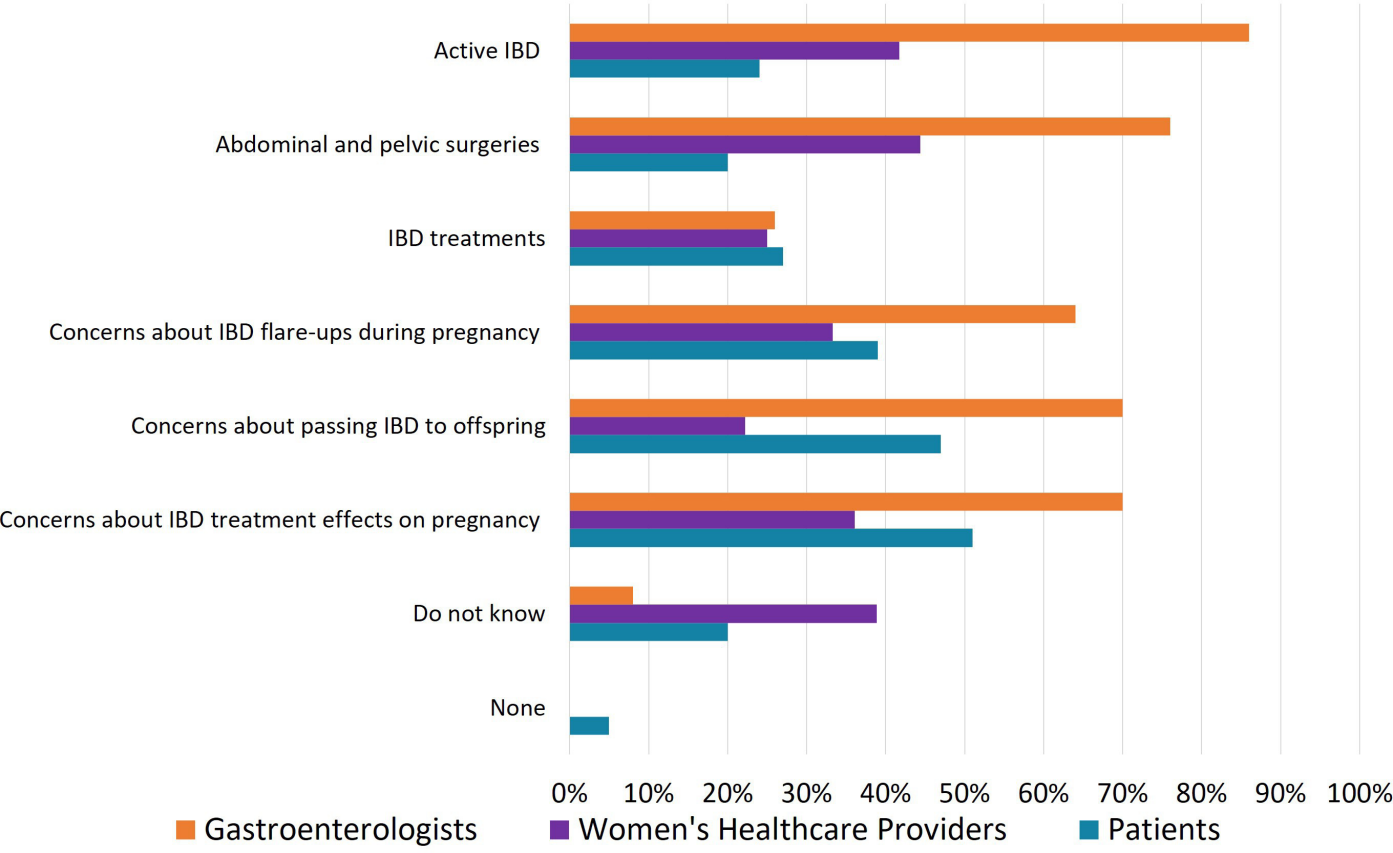
Perceived causes of reduced fertility in women with inflammatory bowel disease (IBD) patient (green), gastroenterologist (orange) and women’s healthcare provider (purple) responses to the question, ‘what do you believe are the causes of reduced fertility in IBD patients?’ (in percentages).

A decreased fertility rate in women with IBD was reported by 33% of patients, 43.1% of WHPs and 84% of GEs. The primary reasons cited by patients included concerns about the impact of IBD medications on pregnancy (51%), fear of transmitting IBD to offspring (47%) and fear of disease flare-ups during pregnancy (39%). Less frequently, patients identified active IBD (24%) and previous pelvic or abdominal surgeries (20%) as contributing factors to decreased fertility. In contrast, GEs and WHPs pointed to active IBD (86% of GEs, 41.7% of WHPs) and past pelvic or abdominal surgeries (76% of GEs, 41.7% of WHPs) as the main reasons for reduced fertility in women with IBD. Detailed results are provided in figure 4.

Thirteen patients (13%), 11 GEs (22%) and nine WHPs (12.5%) believed that in vitro fertilisation (IVF) success rates were reduced in the context of IBD. However, the majority reported uncertainty regarding the impact of IBD on IVF outcomes (80% of patients, 58% of GEs and 70.8% of WHPs). No significant differences were found between the responses of WHPs who had experience caring for women with IBD and those who did not (detailed comparisons are provided in online supplemental material 5).

## Discussion

The findings of this study highlight significant gaps in the fertility and contraception knowledge among patients with IBD, WHPs and GEs. The low rate of patient counselling on fertility outcomes, previously noted in the literature, was confirmed.^7^ This under-counselling has considerable implications, potentially increasing voluntary childlessness, despite evidence indicating that prepregnancy counselling can enhance pregnancy outcomes and reduce risks for adverse obstetric and foetal events.^9 15^

Aboubakr *et al* reported that IBD patients prioritise receiving information on medication safety during pregnancy, disease control and its impact on pregnancy as key components of preconception counselling.^16^ This concern is justified, as active IBD and previous pelvic surgeries are linked to decreased fertility.^17^ Importantly, the risk of active disease during pregnancy is mitigated when remission is achieved before conception, emphasising the necessity for preconception counselling.^18^ Current ECCO guidelines classify most IBD medications, excluding methotrexate, S1P modulators and JAK inhibitors, as low-risk during pregnancy.^3^ The PIANO study supports this, showing no increase in adverse maternal or foetal outcomes (at birth and in the first years of life) linked to the use of thiopurines, anti-tumour necrosis factor alpha agents, vedolizumab or ustekinumab during pregnancy.^19 20^ Additionally, most IBD medications do not negatively impact fertility.^3^ Although the risk of IBD transmission to offspring is slightly elevated compared with the general population, it remains below 5%.^21^ Disseminating these reassuring data is essential for reducing voluntary childlessness due to misconceptions.

Our results reveal a significant discrepancy between the perceptions of patients, WHPs and GEs regarding fertility in women with IBD. This variation may reflect differences in how fertility is defined and understood across these groups. Patients may be more focused on psychological and social factors, such as the decision to remain child-free, whereas healthcare professionals tend to emphasise the physiological factors that contribute to reduced fertility, such as disease activity and previous surgeries. These findings highlight the need for more tailored communication between healthcare providers and patients to address not only the medical aspects of fertility but also the psychological and emotional concerns that may influence a patient’s reproductive decisions.

This study is the first to assess knowledge of contraception and emergency contraception among all key stakeholders involved in contraception choices: women with IBD, WHPs and GEs. Recent findings by Limdi *et al* reported worse outcomes compared with ours, with only 25% of women in a British cohort having discussed reproductive issues with their IBD clinician.^22^ Despite evidence indicating that oral contraceptives have comparable absorption in IBD and healthy women (except those with short bowel syndrome) and do not exacerbate IBD flares, misconceptions were prevalent among WHPs in our cohort.^23^ Notably, oral contraceptives remain the most common choice among women with IBD.^11^ The persistence of misconceptions may stem from reported associations between oral contraceptive use and the onset of IBD (adjusted RR of 1.46 for Crohn's Disease and 1.28 for Ulcerative Colitis after adjustment for smoking).^24^ Although oral contraceptives are not contraindicated, thrombotic risk should be evaluated before initiating combined oestrogen-progesterone pills.^3^ Interestingly, this risk does not appear to be the primary barrier to prescribing oral contraceptives, as they are still frequently prescribed to IBD patients with a high thrombotic risk.^25^

Despite the absence of published data on the impact of IBD on emergency contraception, some respondents indicated that certain methods are contraindicated, and nearly half of WHPs believed that morning-after pills might be less effective. Similarly, misconceptions about IUDs potentially triggering IBD flares or reduced effectiveness were reported.^26^ This might stem from the anatomical proximity of the uterus and rectum and a misperception of ‘local inflammation’ affecting the IUD. Similarly, studies show that women with IBD, including those with ileal pouch-anal anastomosis, have IVF success rates comparable to non-IBD women.^27^

Consistent with the findings of Kashkooli *et al* regarding pregnancy-related IBD knowledge, GEs demonstrated better knowledge on contraception compared with WHPs.^28^ This is concerning, as WHPs are viewed by patients and other healthcare professionals as key sources of information on contraception in our study. Although one might attribute this discrepancy to the fact that approximately one-third of WHPs had never managed women with IBD, our analysis found minimal differences between those who had and those who had not (except for misconceptions about IUDs). In contrast, findings from a British cohort indicated that preferred sources of reproductive counselling for IBD patients were IBD nurses, general practitioners and GEs, rather than gynaecologists.^22^ Moreover, Gawron *et al* previously highlighted that GEs infrequently counsel IBD patients on contraception.^13^ Increasing the commitment of GEs to provide dedicated time for contraception discussions and promoting a multidisciplinary approach that includes targeted training for WHPs are critical to enhancing the quality of IBD patient counselling.

We acknowledge several limitations in this study. First, the survey tool was not previously validated. Although existing tools, such as the Crohn’s and Colitis Pregnancy Knowledge Score, assess pregnancy-related knowledge, they do not address contraception and lack validation for use with healthcare professionals, despite their prior application in this population.^6 29^ Additionally, confounding factors such as prior pregnancy status in patients and the specific number of IBD patients seen by professionals were not controlled for. The sample size limited our ability to assess the effect of CME participation on knowledge. The generalisability of the findings is constrained by potential selection bias, as participants were recruited from a tertiary care centre and may have more severe disease profiles and greater awareness of disease-related complications. The limited response rates further underline this bias as survey respondents may have had a higher baseline interest and knowledge in the topic. Lastly, men with IBD were excluded due to the survey’s focus on female contraception; their inclusion might have revealed even lower levels of understanding about family planning in the general population.^30^

In conclusion, this study underscores the pressing need for IBD-specific CME programmes focusing on contraception and fertility to enhance healthcare professionals’ knowledge. Improved interdisciplinary communication and targeted education for WHPs are necessary to empower IBD patients in making informed reproductive health decisions.

## Supplementary material

10.1136/bmjgast-2024-001669 online supplemental file 1

10.1136/bmjgast-2024-001669 online supplemental file 2

10.1136/bmjgast-2024-001669 online supplemental file 3

10.1136/bmjgast-2024-001669 online supplemental file 4

10.1136/bmjgast-2024-001669 online supplemental file 5

## Data Availability

Data are available upon reasonable request.
