# Supplementary material for: Contraception, fertility and inflammatory bowel disease (IBD): a survey of the perspectives of patients, gastroenterologists and women’s healthcare providers
Source: BMJ Open Gastroenterol. 2025 Mar 16;12(1):e001669. doi: 10.1136/bmjgast-2024-001669 (PMC13059878; doi:10.1136/bmjgast-2024-001669)
Supplement: online supplemental file 2 [file bmjgast-12-1-s002.docx]

**Supplemental data 2: Patients’ survey**

IBD often affects women of childbearing age, and its potential impact on fertility and contraceptive methods has been addressed in recent European guidelines. This anonymous survey aims to assess your understanding of how IBD and its treatments might affect fertility and contraceptive options. Your responses will help us enhance our practices and communication on this topic.

Part 1: Personal Health Profile

1/ What is your age? (years)

2/What is your weight? (kg)

3/ What is your height? (in cm)

4/ What type of IBD do you have? 🞎 Crohn's disease (CD) 🞎 Ulcerative colitis (UC)

🞎 Indeterminate colitis

5/ What is the location and phenotype of your IBD? (Please complete this section with your gastroenterologist if unsure.)

For UC or Indeterminate Colitis: 🞎 E1 (proctitis) 🞎 E2 (left-sided colitis) 🞎 E3 (pancolitis)

For CD: Location: 🞎 L1 (ileal) 🞎 L2 (colonic) 🞎 L3 (ileocolonic) 🞎 L4 (upper GI tract) 🞎 perianal involvement

Phenotype: 🞎 B1 (non stricturing non penetrating) 🞎 B2 (stricturing) 🞎 B3 (penetrating)

6/ When was your IBD diagnosed? (year)

7/ Have you undergone any bowel surgery for IBD? 🞎 Yes 🞎 No

If yes, please specify the type of surgery: 🞎 Small bowel resection 🞎 Partial colectomy

🞎 proctocolectomy with ileal pouch-anal anastomosis 🞎 Total colectomy with ileo-rectal anastomosis

8/ Have you had surgery for perianal lesions? 🞎 Yes 🞎 No

9/ Do you currently consider yourself in remission from IBD? 🞎 Yes 🞎 No

How would you rate your IBD activity level right now? (Scale of 0 to 10, where 0 = not active at all and 10 = extremely active)

🞎 0 🞎 1 🞎 2 🞎 3 🞎 4 🞎 5 🞎 6 🞎 7 🞎 8 🞎 9 🞎 10

10/ What is your smoking status?🞎 Current smoker 🞎 Former smoker 🞎 Non-smoker

11/ Which of the following treatments are you currently receiving or have received in the past? (Please check all that apply)

|  | Current treatment | Past treatment |
| --- | --- | --- |
| None |  |  |
| Amino salicylates (PENTASA® FIVASA® ROWASA®) |  |  |
| Steroids (SOLUPRED® CORTANCYL® BETNESOL®) |  |  |
| Thiopurines (IMUREL®, PURINETHOL®) |  |  |
| Methotrexate (IMETH®, METOJECT®) |  |  |
| Infliximab (REMICADE®, REMSIMA®, INFLECTRA®, FLIXABI®) |  |  |
| Adalimumab (HUMIRA®, AMGEVITA®, IMRALDI®, HULIO®, HYRIMOZ®, IDACIO®, YUFLYMA®, HUKYNDRA®) |  |  |
| Ustekinumab (STELARA®) |  |  |
| Vedolizumab (ENTYVIO®) |  |  |
| Tofacitinib (XELJANZ®) |  |  |
| Others (If yes, specify: ………………………………………………….) |  |  |

12/ Do you have any health conditions other than IBD?

🞎 Skin disease (e.g. psoriasis) 🞎 Primary sclerosing cholangitis 🞎 Joint disease (e.g. ankylosing spondylitis) 🞎 Uveitis

🞎 Others (please specify: ………………………………………………………………………………………………….)

Part 2: IBD, Fertility and Contraception

13/ Have you ever considered how IBD might impact contraceptive options? 🞎 Yes 🞎No

14/ How often has your gastroenterologist discussed the impact of IBD on fertility and contraception with you?

🞎 During a dedicated consultation 🞎 Only when I asked 🞎 At the time of diagnosis

🞎 Once per year 🞎 Once every two years 🞎 Never discussed 🞎 Do not know

15/ Which of the following contraceptive methods might be less effective in patients with IBD, not considering any effects from IBD treatments?

🞎 Progesterone-only pills 🞎 Combined estrogen-progesterone pills

🞎 Contraceptive implants 🞎 Copper intra uterine devices (IUDs) 🞎 Hormonal intra uterine devices (IUDs) 🞎 Vaginal rings and skin patches 🞎 None 🞎 Do not know

16/ Which contraceptive methods, if any, are associated with a risk of IBD flare-ups?

🞎 Progesterone-only pills 🞎 Combined estrogen-progesterone pills

🞎 Contraceptive implants 🞎 Copper intra uterine devices (IUDs) 🞎 Hormonal intra uterine devices (IUDs) 🞎 Vaginal rings and skin patches 🞎 None 🞎 Do not know

17/ Are any emergency contraceptive methods contraindicated for IBD patients, not considering any effects from IBD treatments?

🞎 Morning-after pills 🞎 Intra uterine devices (IUDs) 🞎 None 🞎 Do not know

18/ Which emergency contraceptive methods, if any, may have reduced effectiveness in IBD patients, not considering any effects from IBD treatments?

🞎 Morning after pills 🞎 Intra uterine devices 🞎 None 🞎 Do not know

19/ In your opinion, do IBD patients have a reduced fertility rate?

🞎 Yes 🞎 No 🞎 Do not know

20/ What do you believe are the causes of reduced fertility in IBD patients? (Please select all that apply.)

🞎 Active IBD 🞎 Abdominal and pelvic surgeries 🞎 IBD treatments

🞎 Concerns about IBD flare-ups during pregnancy 🞎 Concerns about passing IBD to offspring

🞎 Concerns about IBD treatment effects on pregnancy 🞎 Do not know

21/ Is the success rate of in vitro fertilization (IVF) for IBD patients similar to that of the general population?

🞎Yes 🞎 No 🞎 Do not know

22/ Which healthcare professional(s) do you think can best address your questions about contraception and fertility? (Please select all that apply.)

🞎 Midwife 🞎 Medical gynecologist 🞎-Obstetrician 🞎 General practitioner

🞎 Gastroenterologist
