# Supplementary material for: Contraception, fertility and inflammatory bowel disease (IBD): a survey of the perspectives of patients, gastroenterologists and women’s healthcare providers
Source: BMJ Open Gastroenterol. 2025 Mar 16;12(1):e001669. doi: 10.1136/bmjgast-2024-001669 (PMC13059878; doi:10.1136/bmjgast-2024-001669)
Supplement: online supplemental file 3 [file bmjgast-12-1-s003.docx]

**Supplemental data 3: Gastroenterologists’ survey**

Inflammatory bowel diseases (IBD) affect women of childbearing age, and recent European guidelines (*J. Torres et al. JCC 2023*) address the impact of IBD and its treatments on fertility and contraceptive methods.

This anonymous survey seeks to understand your knowledge and practices related to these issues. Additional surveys will be administered to gynecologists, obstetricians, midwives, and patients as part of this comprehensive study.

Part 1: Professional Profile

1/ What is your gender?🞎 Male 🞎 Female

2/ How many years have you been practicing medicine?

3/ In what type of setting do you primarily practice?

🞎 General hospital 🞎 Private medical practice🞎 University Hospital

🞎 Both private and public hospital practice (please specify: ………………….)

🞎 Resident at a university hospital

4/ Approximately what percentage of your patient base consists of IBD patients?

5/ Have you participated in any continuing medical education (CME) or training specifically related to contraception, fertility, and IBD?

🞎 Yes 🞎 No

Part 2: Knowledge and Practices on IBD, Fertility, and Contraception

6/ Have you considered the benefit-risk ratio of different contraceptive methods for patients with IBD?

🞎 Yes 🞎No

7/ How often do you discuss fertility and contraception with your IBD patients?

🞎 During a dedicated visit 🞎 Only if the patient inquires about it 🞎 At the time of diagnosis

🞎 Once per year 🞎 Every two years 🞎 Never 🞎 Do not know

8/ Which of the following contraceptive methods might have reduced effectiveness in IBD patients, not considering any effects from IBD treatments?

🞎 Progesterone-only pills 🞎 Combined estrogen-progesterone pills

🞎 Contraceptive implants 🞎 Copper intra uterine devices (IUDs) 🞎 Hormonal intra uterine devices (IUDs) 🞎 Vaginal rings and skin patches 🞎 None 🞎 Do not know

9/ Which contraceptive methods, if any, are associated with a risk of triggering IBD flare-ups?

🞎 Progesterone-only pills 🞎 Combined estrogen-progesterone pills

🞎 Contraceptive implants 🞎 Copper intra uterine devices (IUDs) 🞎 Hormonal intra uterine devices (IUDs) 🞎 Vaginal rings and skin patches 🞎 None 🞎 Do not know

10/ Which emergency contraceptive methods, if any, are contraindicated in IBD patients, not considering any effects from IBD treatments?

🞎 Morning-after pills 🞎 Intra uterine devices 🞎 None 🞎 Do not know

11/ Which emergency contraceptive methods might be less effective in IBD patients, not considering any effects from IBD treatments?

🞎 Morning-after pills 🞎 Intra uterine devices 🞎 None 🞎 Do not know

12/ In your opinion, do IBD patients have a reduced fertility rate?

🞎 Yes 🞎 No 🞎 Do not know

13/ What do you believe are the causes of reduced fertility in IBD patients? (Please select all that apply.)

🞎 Active IBD 🞎 Abdominal and pelvic surgeries 🞎 IBD treatments

🞎 Concerns about IBD flare-ups during pregnancy 🞎 Concerns about passing IBD to offspring

🞎 Concerns about IBD treatment effects on pregnancy 🞎 Do not know

14/ Is the success rate of in vitro fertilization (IVF) for IBD patients similar to that of the general population?

🞎Yes 🞎 No 🞎 Do not know

15/ In your opinion, which healthcare professionals are best suited to answer IBD patients’ questions about contraception and fertility? *(Please select all that apply.)*

🞎 Midwife 🞎 Medical gynecologist 🞎-Obstetrician 🞎 General practitioner

🞎 Gastroenterologist
