# Supplementary material for: Contraception, fertility and inflammatory bowel disease (IBD): a survey of the perspectives of patients, gastroenterologists and women’s healthcare providers
Source: BMJ Open Gastroenterol. 2025 Mar 16;12(1):e001669. doi: 10.1136/bmjgast-2024-001669 (PMC13059878; doi:10.1136/bmjgast-2024-001669)
Supplement: online supplemental file 5 [file bmjgast-12-1-s005.docx]

**Supplementary Table 1: Comparison of Women Healthcare Providers (WHPs) based on Experience in Managing Women with IBD**

|  | WHPs with no experience in managing IBD patients (n=22) | WHPs with experience in managing IBD patients (n=50) | p-value |
| --- | --- | --- | --- |
| Participation in any continuing medical education (CME) or training specifically related to contraception, fertility, and IBD | 0 (0) | 2 (4) | 0.341 |
| Consideration of the benefit-risk ratio of different contraceptive methods for patients with IBD | 11 (50) | 21 (42) | 0.529 |
| Contraceptive methods that might have reduced effectiveness in IBD patients | | | |
| Progesterone-only pills | 14 (63.6) | 23 (46) | 0.168 |
| Combined estrogen-progesterone pills | 14 (63.6) | 25 (50) | 0.489 |
| Contraceptive implants | 1 (4.5) | 0 | 0.129 |
| Copper intra uterine devices (IUDs) | 2 (9.1) | 0 | 0.031 |
| Hormonal IUDs | 1 (4.5) | 0 | 0.129 |
| Vaginal rings and skin patches | 2 (9.1) | 0 | 0.031 |
| Contraceptive methods associated with a risk of triggering IBD flare-ups | | | |
| Progesterone-only pills | 1 (4.5) | 3 (6) | 0.804 |
| Combined estrogen-progesterone pills | 5 (22.7) | 12 (24) | 0.907 |
| Contraceptive implants | 0 | 0 | / |
| Copper IUDs | 9 (40.9) | 5 (10) | 0.002 |
| Hormonal IUDs | 2 (9.1) | 1 (2) | 0.165 |
| Vaginal rings and skin patches | 3 (13.6) | 4 (8) | 0.618 |
| Emergency contraceptive methods contraindicated in IBD patients | | | |
| Morning-after pills | 1 (4.5) | 5 (10) | 0.44 |
| IUDs | 7 (31.8) | 1 (2) | < 0.001 |
| Emergency contraceptive methods less effective in IBD patients | | | |
| Morning-after pills | 15 (68.2) | 19 (38) | 0.018 |
| IUDs | 0 | 1 (2) | 0.504 |
| In your opinion, do IBD patients have a reduced fertility rate? | | | 0.157 |
| Yes | 8 (36.4) | 23 (46) |  |
| No | 0 | 5 (10) |  |
| Do not know | 14 (63.6) | 22 (44) |  |
| Causes of reduced fertility in IBD patients? | | | |
| Active IBD | 9 (40.9) | 21 (42) | 0.931 |
| Abdominal and pelvic surgeries | 11 (50) | 21 (42) | 0.529 |
| IBD treatments | 5 (22.7) | 13 (26) | 0.768 |
| Concerns about IBD flare-ups during pregnancy | 9 (40.9) | 15 (30) | 0.366 |
| Concerns about passing IBD to offspring | 7 (31.8) | 9 (18) | 0.194 |
| Concerns about IBD treatment effects on pregnancy | 10 (45.5) | 16 (32) | 0.274 |
| Is the success rate of in vitro fertilization (IVF) for IBD patients similar to that of the general population? | | | 0.721 |
| Yes | 2 (9.1) | 7 (14) |  |
| No | 3 (13.6) | 9 (18) |  |
| Do not know | 17 (77.3) | 34 (68) |  |

Results are expressed as n(%) and compared by a chi-squared test (or Fisher’s exact tests when appropriate).
